# Flawed Reasoning Allows the Persistence of Mainstream Atherothrombosis Theory

**DOI:** 10.7759/cureus.2377

**Published:** 2018-03-27

**Authors:** Gregory D Sloop, Gheorghe Pop, Joseph J Weidman, John A St. Cyr

**Affiliations:** 1 N/A, Independent Researcher; 2 Cardiology, Radboud University Nijmegen Medical Center, Nijmegen, The Netherlands, Nijmegen, NLD; 3 Research and Development, Jacqmar, Inc., Minneapolis, USA

**Keywords:** atherothrombosis, paradigm shift, theory, lipid, cholesterol, logic, ad hoc, kuhn, popper, inflammation

## Abstract

Deaths due to atherothrombosis are increasing throughout the world except in the lowest socio-demographic stratum. This is despite 60 years of study and expenditure of billions of dollars on lipid theory. Nevertheless, mainstream atherothrombosis theory persists even though it has failed numerous tests. Contrary data are ignored, consistent with the practice of science as envisioned by Thomas Kuhn. This paper examines defects in mainstream atherogenesis theory and the flawed logic which allows its persistence in the face of what should be obvious shortcomings.

## Introduction and background

Charles Sydney Burwell, M.D., Dean of Harvard Medical School from 1935 to 1949, famously said, “Half of what you are taught as medical students will in 10 years have been shown to be wrong" [1]. Most medical students in the United States have heard this quote at least once. This statement is supported by a study of 363 papers published in the New England Journal of Medicine between the years 2001 and 2010 which found that 40.2% reversed a medical practice while 38.0% reaffirmed it [2]. The fluid nature of best practice begs the question, “Why is the medical mainstream resistant to a paradigm shift?” Informed by Thomas Kuhn’s theory of paradigm shifts, historian of science Marc De Mey wrot,e “As long as paradigm-compatible findings keep accumulating, it is rather easy to ignore or suppress discordant data as irrelevant or just noisy elements [3].”

Dismissing discordant data is facilitated by unquestioned acceptance of a flawed theory and faulty logic which is rarely recognized as such. In this review, the authors will first examine the enlarging body of evidence which refutes lipid and inflammatory theories of atherothrombosis. Then the authors examine the erroneous reasoning which perpetuates belief in mainstream thought despite considerable conflicting evidence. Although this review focuses on atherothrombosis, these errors can lead researchers astray and hinder progress in any field. 

**Figure 1:**
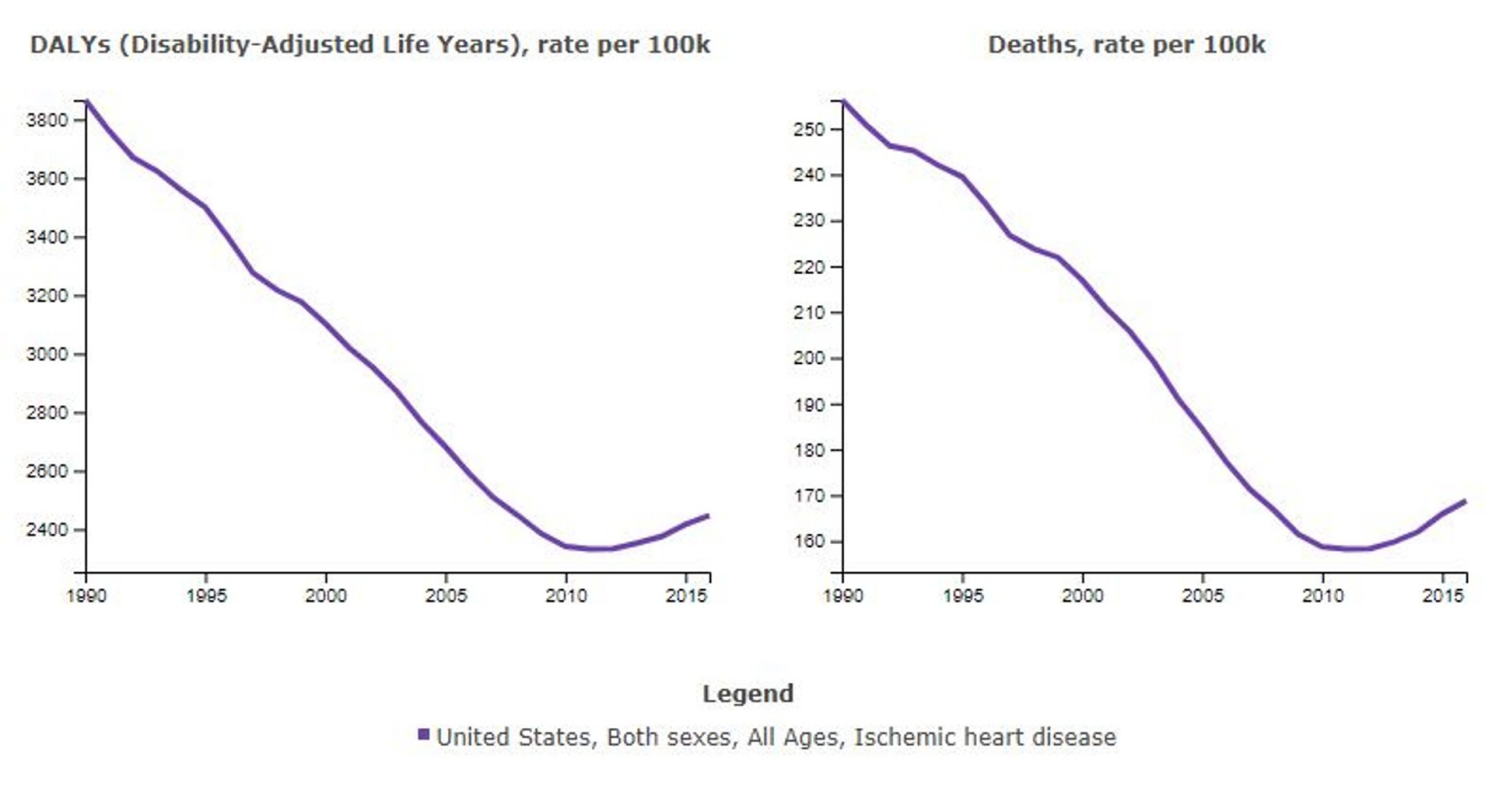
Ischemic Heart Disease in the U.S. Disability-adjusted life years lost (DALYs) per 100,000 due to ischemic heart disease in the United States. Rates began to increase in 2011. Courtesy of the Institute for Health Metrics and Evaluation.

**Figure 2:**
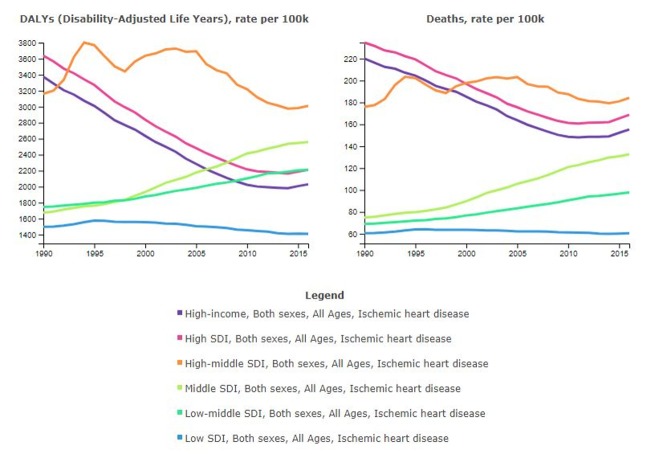
Global Morbidity and Mortality Due to Ischemic Heart Disease Disability-adjusted life years (DALYs) per 100,000 due to ischemic heart disease stratified by socio-demographic index (; a measure of per capita income, average educational attainment, and fertility rate and in high income countries from 1990 to 2016. Rates for high income and high and high-mid socio-demographic index countries decreased from approximately 2005 until roughly 2010 and then began to increase in approximately 2014. No increase was noted in the low socio-demographic stratum. Courtesy of the Institute for Health Metrics and Evaluation.

Despite 60 years of study and an investment of billions of dollars, the rate of deaths due to coronary artery disease is increasing in the United States and in all other countries except the lowest socio-demographic populations worldwide (Figure 1, Figure 2) [4].

Deaths due to heart disease increased by 3% in the US between 2014 and 2015, the latest years for which data are available [5]. Clearly, lipid theory is not powerful enough to provide the insight necessary to control atherothrombosis.

## Review

Mainstream atherothrombosis theory has failed numerous tests

The essence of lipid or mainstream atherothrombosis theory is that cholesterol, cholesterol ester, or some other component of low density lipoprotein (LDL) accumulates in the arterial wall and elicits an inflammatory response which culminates in an atherosclerotic plaque. Chronic oxidative stress is felt to be necessary to modify LDL so that it can be phagocytized by macrophages via the scavenger receptor. High density lipoprotein (HDL) is felt to protect against atherothrombosis by removing cholesterol from the periphery by "reverse transport" and returning it to the liver for catabolism.

Antioxidant therapy, including Vitamin E supplementation, failed to alter cardiovascular risk even though vitamin E prevents oxidative modification of LDL in vitro [6]. Inhibitors of cholesteryl ester transfer protein (CETP), which raise HDL levels, failed to decrease cardiovascular events in the following trials: the ILLUMINATE (Investigation of Lipid Level Management to Understand its Impact in Atherosclerotic Events) trial using torcetrapib, the dal-OUTCOMES trial assessing dalcetrapib, and the ACCELERATE (Assessment of clinical effects of cholesteryl ester transfer protein inhibition with evacetrapib in patients at high risk for vascular outcomes) trial with evacetrapib. The failure of evacetrapib was particularly surprising to the mainstream cardiology community because HDL-cholesterol levels increased by 128% and LDL-cholesterol levels decreased by 35% in patients with an elevated risk for cardiovascular disease [7]. The CETP inhibitors increase the size and number of HDL particles which will increase blood viscosity [8]. The marginal efficacy of cholesterol-lowering therapy was shown by Kristensen et al., who found that primary prevention with statin therapy resulted in a median postponement of death by 3.2 days, and only 4.1 days in secondary prevention trials [9]. Finally, and most significantly, DuBroff reported that 44 prospective, randomized controlled studies of cholesterol-lowering therapy, three of which involved diet, showed no mortality benefit despite reductions in LDL-cholesterol levels of up to 50%. Additionally, 30 of these studies showed no reduction in cardiovascular events [10].

Two trials of anti-inflammatory therapy failed to show any benefit in preventing cardiovascular disease. Losmapimod, which inhibits p38 mitogen-activated protein kinase, an enzyme which amplifies the inflammatory cascade through enhanced production of TNF-α, IL-6, and other cytokines, failed to improve outcome in the LATITUDE-TIMI 60 [Losmapimod to Inhibit p38 MAP (mitogen activated protein) Kinase as a Therapeutic Target and Modify Outcomes after an Acute Coronary Syndrome-Thrombolysis in Myocardial Infarction 60] study [11]. Lipoprotein-associated protein kinase is an enzyme hypothesized to cause atherogenesis and contribute to plaque instability through pathways related to inflammation. Darapladib, an inhibitor of this enzyme, failed to modify outcome in the SOLID-TIMI 52 (Stabilization of pLaques Using Darapladib-Thrombolysis in Myocardial Infarction 52) trial [12]. The persistence of mainstream atherothrombosis theory despite these negative results substantiate Kuhn’s assertion that discordant findings are ignored by the mainstream.

The results of the recent CANTOS (Canakinumab Anti-Inflammatory Thrombosis Outcomes Study) trial will be discussed below.

Kuhnian dynamics in action

Dietary cholesterol was thought to play a role in atherothrombosis since Anitschskow induced arterial lesions in rabbits by feeding them massive amounts of cholesterol. The observation by Keys et al. that heart disease was more common in well-nourished businessmen than in the malnourished population of World War II Europe, coupled with his 1953 report in which the only variable that was higher in businessmen who suffered from heart disease was serum cholesterol [13], established lipid theory as the mainstream paradigm.

Since the 1960s until recently, guidelines recommended limiting cholesterol consumption. Now, overconsumption of cholesterol is recognized as not a cause for concern [14]. In 2014, McNamara noted that changing the recommendations about cholesterol consumption was a long, contentious process despite evidence which convinced objective nutritionists. He noted that authorities in some countries experienced cognitive dissonance (i.e., performing an action which is contrary to personal beliefs) and continued to promote outdated and potentially hazardous dietary recommendation based on a refuted hypothesis [15].

There are many reasons for resistance to a paradigm shift. Scientists are not as dispassionate, objective, and rational in processing information as computers. It is probably difficult for some authorities to reverse a long-held position and admit that their previous advice was incorrect. This may be especially true for clinicians who depend on their authority to encourage compliance with their recommendations, and who might fear that a change in a long held position will damage their credibility. The same may apply to prestigious institutions such as the American Heart Association (AHA) and National Heart, Lung, and Blood Institute (NHLBI), which are composed of numerous individual authorities. The imprimatur of these organizations helped entrench lipid theory. Of particular importance in this regard is the National Cholesterol Education Program (NCEP), a program of the NHLBI which has activities aimed at the public. In addition, busy clinicians may not feel they have time to spend on a new theory which may not stand the test of time, or enthusiasm for assimilating a new paradigm after working an 18-hour day. It is not surprising that the physicist Max Plank wrote "a new scientific truth does not triumph by convincing its opponents and making them see the light, but rather because its opponents eventually die, and a new generation grows up that is familiar with it."

Conceptual errors surrounding mainstream atherothrombosis theory

In 2002, Professor Emeritus P.J. Scott of the University of Auckland, New Zealand, wrote: “Those who may seek now, or in the future, to denigrate the achievements of medical science in the 20th century will find numerous examples within the general field of atherosclerosis research of oversimplification, extrapolation, and near total abandonment of the principles of the scientific method" [16]. We address these issues in the next sections.

Lipid Theory is Incomplete

An incomplete theory is one which explains only some, not all outcomes. It does not mean that the theory is wrong, but it should not be considered as the final word. For example, Newtonian mechanics is incapable of explaining the precession of the perihelion of planet Mercury, an anomaly noted in 1859. However, the utility of the Newtonian laws of motion has never been questioned because they have always provided useful results. It was only when Einstein introduced the theory of general relativity that Mercury’s orbit was completely understood. General relativity is a more complete theory.

Lipid theory is incomplete. It cannot explain the accelerated atherogenesis (i.e., development of an atherosclerotic plaque) associated with ageing, hypertension, diabetes mellitus, cigarette smoking, exposure to fine particulate matter air pollution, hypercoagulability, hypofibrinolysis, hyperfibrinogenemia, menopause, male gender, erythropoiesis stimulating agents, deep venous thrombosis, elevated blood viscosity, elevated sedimentation rate, decreased erythrocyte deformability, consumption of the Western diet and trans fats, lupus anticoagulant, Protein C and S deficiencies, nephrotic syndrome, splenectomy, or sticky platelet syndrome. It also is inadequate to explain the decreased atherogenesis associated with aspirin use and clotting disorders such as von Willebrand disease and hemophilias. It should be emphasized that the hematologic conditions in these lists affect the development of atherosclerotic plaques, which are organized mural thrombi, as well as superimposed thrombosis [17-19]. References demonstrating that hematologic disorders can cause atherogenesis are provided in the Appendix. Further evidence of the limitation of lipid theory is that only 30,000 of the estimated 100,000 excess annual myocardial infarctions in the US attributable to the consumption of trans fats could be explained by lipid abnormalities [20]. Inflammatory theory is incomplete because it cannot explain the localization of atherosclerotic plaques. 

Extrapolation

Extrapolation is the extension of the findings from a particular case to make a generalization about all cases. Progress in atherothrombosis research is hindered by two examples of incorrect extrapolation. First, extrapolating from familial hypercholesterolemia to conclude that cholesterol causes atherothrombosis is incorrect. Second, the theory that atherothrombosis is an inflammatory disease based on the accelerated disease in rheumatoid arthritis and other true inflammatory diseases is also incorrect. The theories that hypercholesterolemia and chronic inflammation cause atherothrombosis are limited or special cases of the broader theory that elevated blood viscosity accelerates atherogenesis by promoting mural thrombosis [17-19]. Discussion of the role of elevated blood viscosity in atherothrombosis, metabolic syndrome, diabetes mellitus type II, and slight elevations of C-reactive protein are beyond the scope of this review. The interested reader is directed to references 17-19.

The recent CANTOS trial has been touted as a test of the inflammatory etiology of atherothrombosis. Subjects who received 150 mg of canakinumab, a monoclonal antibody against interleukin-1b, had a 14% decrease in myocardial infarction, stroke or fatal cardiovascular event after 3.7 years [hazard ratio: 0.85 (95% CI, 0.74 to 0.98; P = 0.021)] [21]. Importantly, in a phase IIb trial, canakinumab therapy decreased plasma fibrinogen levels 21%, from 330 mg/dL to 260 mg/dL, both normal values [22]. Fibrinogen is an independent risk factor for atherothrombosis and increases blood viscosity. Therefore, it is possible that the benefit of canakinumab therapy is due to decreased fibrinogen concentrations and blood viscosity, not due to decreased inflammation. Unfortunately, fibrinogen concentrations were not reported in Phase III of the CANTOS study.

The accelerated atherothrombosis associated with true chronic inflammatory diseases can be explained by increased blood viscosity due to hyperfibrinogenemia, hypergammaglobulinemia, and immune complexes. Fibrinogen augments erythrocyte aggregation, which elevates the erythrocyte sedimentation rate and blood viscosity at low shear rates. IgM molecules are also large enough to foster erythrocyte aggregation. Increased protein concentrations from any cause will increase plasma viscosity and may foster erythrocyte aggregation via the “depletion” force. This force drives erythrocytes together when protein concentrations surrounding erythrocytes are greater than that found between them.

Falsification and Falsifiability Are Ignored

The renowned philosopher of science Sir Karl Popper felt that the essence of a scientific theory is that it should be falsifiable. A scientific theory should make specific predictions which can be tested. A theory is refuted if testing fails to confirm these predictions. Popper also wrote “It is easy to obtain confirmations, or verifications, for nearly every theory—if we look for confirmations" [23]. Thus, in the scientific method as conceptualized by Popper, a theory can only be disproved, not proved. No number of confirmations can ever prove a theory.

Popper’s work sheds light on studies of statin therapy. While statins have been shown to reduce morbidity and mortality from atherothrombosis in some studies, DuBroff’s work shows that they have failed in a substantial number of studies despite significantly decreasing LDL levels. This failure falsifies the theory that elevated LDL or cholesterol is the cause of atherothrombosis. However, some positive results of statin therapy are expected because high LDL levels can increase blood viscosity, as in familial hypercholesterolemia. Data from the Framingham study show that only 27% of attributable risk for coronary heart disease in men and 34% in women are due to hypercholesterolemia [24]. In the Copenhagen Heart Study, hypercholesterolemia was the sixth strongest variable in determining the relative risk of coronary heart disease [25]. Data collected by the Asia Pacific Studies Collaboration showed that the fraction of fatal coronary heart disease attributable to high cholesterol ranged from 0 to 14% in the 16 reporting counties [26]. These data are consistent with the theory that LDL is only one of the multiple contributors to elevated blood viscosity and atherothrombosis. Further, elevated LDL concentrations are a weak risk factor, resulting in a large number of failed trials. Because a minority of cases of atherothrombosis is caused by elevated LDL, a positive outcome is expected in some but not all studies of statin use in atherothrombosis.

Apologists for lipid theory consistently claim that lower concentrations of LDL are better for preventing atherothrombosis. In dismissing a negative result, apologists can always argue that cholesterol was not reduced sufficiently. By this argument, excluding LDL as the cause of atherothrombosis will require finding the disease in someone with no lifetime exposure to LDL, clearly an impossibility. Excluding cholesterol as the cause is also impossible because it is a component of all animal cell membranes. For all practical purposes, these arguments render the notion that LDL or cholesterol is the cause of atherothrombosis nonfalsifiable and unscientific. Excluding LDL or cholesterol as the cause is a Sisyphean task at best and unnecessary by the scientific method, which only requires refutation.

The cholesterol which is thought to elicit the chronic inflammation that causes atherothrombosis is contained within foam cells in fatty streaks. Even the mainstream recognizes that for unknown reasons only some fatty streaks progress, yet another non-falsifiable belief. In this case, mainstream atherothrombosis theory persists because a particular segment of artery can be studied histopathologically only once. Fatty streaks are flat and cannot be detected radiologically.

Clinical trials of antioxidant vitamins and immunohistopathology refute oxidation theory. Immunohistopathology shows that oxidized LDL does not cause monocyte chemotaxis, cytotoxicity, or apoptosis as suggested by in vitro studies [27]. The persistent belief in the role of chronic oxidative stress in atherothrombosis can be attributed to the dominance of lipid theory and non-falsifiable defense of the oxidation hypothesis. After refutation in clinical trials, two prominent proponents of the oxidation hypothesis wrote:

“The hypothesis that oxidative modification of LDL plays a significant role in atherogenesis in humans is not necessarily disproved by the failure of these particular clinical trials any more than a negative trial of an ineffectual antibiotic in Pneumococcal pneumonia would prove that pneumonia is not a bacterial disease. The oxidative modification hypothesis is not that vitamin E will ameliorate the human disease but that oxidative modification of LDL and/or other oxidative events play a significant role in human atherogenesis as it does in animal models of atherogenesis. A corollary of the hypothesis is that some appropriate antioxidant intervention, at some appropriate dosage, in appropriately selected patients over an appropriate time interval has the potential to improve prognosis [28]. 

This paragraph contains numerous non-falsifiable statements meant to prevent refutation of oxidation theory, which is an ad hoc modification of lipid theory. Obviously, no observations are excluded by these vague statements. As weak as it is, such an argument is sufficient to defend an accepted hypothesis. To regain standing as a scientific hypothesis, a specific falsifiable statement about oxidation theory must be made.

Ad Hoc Modifications

Ad hoc modifications are changes to a hypothesis which prevent its refutation. Sir Karl Popper held a negative view about ad hoc modifications, writing “Such a procedure is always possible, but it rescues the theory from refutation only at the price of destroying, or at least lowering, its scientific status [23].” Oxidation theory is an ad hoc modification made necessary when it was shown that uptake of cholesterol by the LDL receptor was insufficient to form a macrophage foam cell because of negative feedback. However, chemically-modified LDL can be taken up by the scavenger receptor in sufficient quantities to create a foam cell. Oxidation theory has been refuted as discussed above.

Reverse transport of cholesterol is an ad hoc modification added to explain the protective effect of HDL. Unmodified reverse transport theory is refuted by the rarity of atherothrombosis in some subjects with extremely low levels of HDL due to lecithin:cholesterol acyltransferase deficiency [29] and the failure CETP inhibitors, which elevate HDL levels. This has led to yet another ad hoc modification, “dysfunctional HDL.” Some in vitro evidence has been offered in support of this belief. These data should be treated with skepticism because it has been shown very recently that both increased and decreased activity of putative markers of HDL function can be seen when macrophages are incubated with HDL that was not stored in the presence of cryoprotectants [30]. For the belief in HDL function to qualify as a hypothesis, a specific molecular defect in dysfunctional HDL should be identified and studied prospectively to determine if this defect increases the risk of atherothrombosis. 

There are several possible reasons for the uncritical acceptance of lipid theory. The endorsement of the theory by funding organizations like the NHLBI and AHA is a conspicuous reason. Lack of training in the scientific method is another possibility. Most scientists are aware that accepting a p value of 0.05 as significant means that there is a 5% chance of incorrectly rejecting the null hypothesis. Thus, someone under the impression that discordant finds are rare may dismiss them as statistical outliers and not shortcomings of the theory. The work of DuBroff et al. [10] shows that discordant data are too common to be dismissed and must be considered a shortcoming in lipid theory. 

Fallacies which perpetuate acceptance of mainstream atherothrombosis theory

The next section is inspired by Follies & Fallacies in Medicine by Petr Skrabanek and James McCormick [31]. 

The “Fallacy of Association Being Causal” and the “Fallacy of the Simple Explanation”

Although most physicians and scientists know causality cannot be proven from an association, it is not clear that this knowledge is exercised as widely as it should be. For example, hypercholesterolemia or elevated plasma LDL concentrations are widely assumed to cause atherothrombosis, and slight elevations of C-reactive protein are assumed to make atherothrombosis an inflammatory disease. Rather than falling into the trap of accepting causality when an association is noted between two common conditions, prudence, caution, and intellectual rigor should prompt a search for a factor which causes both, such as elevated blood viscosity [20]. Physicians are not the only ones who may accept an idea because it offers a simple solution to a complex problem. This urge should be tempered because as H.L. Menken observed, “for every complex problem there is a solution that is simple, direct, and wrong.” 

The “Weight-of-evidence Fallacy”

To date, germ theory, general relativity, cell theory, heliocentric theory, and plate tectonics are supported by all available data, not just the bulk. Obviously, explaining all observations should be the goal of every theory. By this standard, lipid theory is clearly wanting. The “weight-of-evidence fallacy” refers to the belief that the majority of evidence determines truth. This fallacy is widely used to support lipid theory, even appearing in the title of lipidologist Daniel Steinberg’s defense of lipid theory, The Cholesterol Wars: Cholesterol Skeptics vs. the Bulk of Evidence. This fallacy is also seen in the following quotation:

“The suggestion that the ‘limited success of cholesterol-lowering therapy in numerous prospective randomized controlled studies, some of which show significant decreases in serum LDL cholesterol but no improvement in outcome’ refutes the causal effect of LDL on the risk of ASCVD [atherosclerotic cardiovascular disease] is not a quantitatively literate argument. Instead, a synthesis of the totality of the evidence … provides overwhelming quantitative evidence that LDL causes ASCVD… [32].”

Regarding the weight of evidence fallacy, Skrabanek and McCormick wrote: “Such an approach to establishing truth is nonscience: not only is it nonscience; it is also dangerous, because reasoning of this sort may lead to action that (particularly in the field of preventive medicine) can touch many people's lives [31].” The danger of accepting lipid theory is that it makes the search for a better theory unnecessary.

Awareness of certain ideas may have suggested that a preponderance of evidence is sufficient to determine truth, or that eliminating discrepant observations is impossible. Clinicians and researchers are aware of biological variability. Because of biological variability, not all humans respond to an intervention in the same way. Further, an individual patient will not react as predictably or reproducibly as a molecule will. The concept of fuzzy logic became widely accepted in the 1970s. According to fuzzy logic, values of truth exist as a continuum between completely true and completely false. Awareness of this concept, misinterpreted and misapplied, could make workers comfortable with accepting a preponderance of data as determining truth. The Heisenberg uncertainty principle states that there is a finite limit to which the precision of certain pairs of variables, such as position and momentum, can be known. The concept of a limit to precision could also make workers comfortable with settling for a preponderance of data to determine truth. Finally, the Copenhagen interpretation of quantum mechanics is typically presented to the public by non-physicists as “a cat can be both dead and alive at the same time.” This concept, in conjunction with the uncertainty principle, could foster the notion that some phenomenon cannot be completely known, making a preponderance of data perhaps the best scientists can hope for.

There are three major reasons why judging truth by the weight of evidence can lead to the wrong conclusion. First, the majority of researchers, by definition, are always working within the mainstream paradigm. These workers are unlikely to boldly question the mainstream paradigm. Further, they are likely to interpret experimental findings within the framework of the mainstream paradigm, not in a different light. Second, it is easy to obtain confirmations for nearly every theory, as noted by Popper. Finally, it is possible to misinterpret even a large body of evidence, as was the case in Eijkman’s theory of the cause of beriberi.

Christiaan Eijkman won the Nobel Prize in 1929 for his discovery of thiamine while investigating the cause of beriberi. Eijkman initially hypothesized that beriberi was caused by a microbe, influenced by the newly discovered germ theory and his training with the great bacteriologist Robert Koch. At that time, some physicians thought all disease might be caused by germs. Further, the pattern of outbreaks, involving housing such as prisons, ships, insane asylums, and impoverished neighborhoods, suggested contagion through poor hygiene. When Eijkman noticed that chickens fed with polished rice developed beriberi and recovered when fed unpolished rice, he came to believe that rice grains contained an antitoxin and that the rice sheath contained the antitoxin [33]. To test his hypothesis, he studied over 300,000 prisoners at 101 sites, and ruled out other factors which might be vectors for infectious disease. The data showed that consumption of unpolished rice prevented beriberi, and seemed to confirm Eijkman’s hypothesis. The disease was subsequently controlled by changing diets. In his paper, "The Epistemology of Error", Douglas Allchin wrote: "Though Eijkman’s conclusions fit the evidence, they were not necessarily free from error. Other interpretations, outside Eijkman’s conceptual horizon, were also possible. Eijkman’s successor in Java, Gerrit Grijns, saw the opposite gestalt: namely, something was missing rather than something present. … Contrary explanations, here, each fit the available evidence [34].” 

Eijkman’s work resulted in the control of beriberi, even though his hypothesis about its cause was incorrect. A massive study did not correct the mistake. Parallels between the research into the cause of beriberi and the current state of atherothrombosis research include more than one explanation which can explain the available evidence, a large-scale study which seems to confirm the erroneous hypothesis (multiple studies in the case of atherothrombosis) and a true explanation which is beyond the conceptual horizon of most. Even though the true cause of atherothrombosis (i.e., promotion of mural thrombosis) is not recognized by the mainstream, extrapolation from a special case (familial hypercholesterolemia) has led to a modestly successful therapy. 

Given the resolve, resources, and an understanding of its pathogenesis, a disease can be controlled. Witness the successes in controlling scurvy, polio, and hookworm disease. In the case of atherothrombosis, the resolve and resources are present, but the understanding is lacking. The limited success of lipid theory does not mean that atherothrombosis is an exception, or beyond our ken. It merely shows that lipid theory is incomplete. It is time for a paradigm shift.

## Conclusions

In spite of extensive investigation into the role of lipids in atherothrombosis, the disease is not controlled and death rates are increasing around the globe. This failure is due to shortcomings of lipid theory. Lipid theory is incomplete because it does not explain the majority of cases of atherothrombosis. The theory that hypercholesterolemia is the cause of atherothrombosis and the theory that atherothrombosis is an inflammatory disease are examples of inappropriate extrapolation. These theories are special cases of the general theory that increased viscosity accelerates atherothrombosis by fostering mural thrombosis. Control of atherothrombosis will require that lipid theory is superseded by a superior theory.

## Appendices

Bibliography of publications examining the effect of hematologic conditions and drugs on atherogenesis

Deep Venous Thrombosis

P. Prandoni, F. Bilora, A. Marchiori A et al., “An association between atherosclerosis and venous thrombosis,” New England Journal of Medicine vol. 348, no. 15, pp. 1435-41, 2003.

W. Ageno, C. Becattini, T. Brighton et al., “Cardiovascular risk factors and venous thromboembolism: a meta-analysis,” Circulation vol. 117, no. 1, pp. 93-102, 2008.

Y. Mi, S. Yan, Y. Lu et al., “Venous thromboembolism has the same risk factors as atherosclerosis: a PRISMA-compliant systemic review and meta-analysis,” Medicine (Baltimore) vol. 95, no. 32, e4495 doi: 10.1097/MD.0000000000004495, 2016.

P. Prandoni, “Venous and arterial thrombosis: two aspects of the same disease?” Clinical Epidemiology, vol. 1, no. 1, pp. 1-16, 2009. 

P. Prandoni, “Venous and arterial thrombosis: is there a link?” Advances in Experimental Medicine and Biology, vol. 906, pp. 273-83, 2017.

T. Kawasaki, J. Kambayashi and M. Sakon, “Hyperlipidemia: a novel etiologic factor for deep venous thrombosis,” Thrombosis Research, vol. 79, no. 2, pp. 147-51, 1995. 

Elevated Blood Viscosity

J.A. Dormandy and J.B. Edelman, “High blood viscosity: an aetiologic factor in venous thrombosis,” British Journal of Surgery vol. 60, no. 3, pp. 197-90, 1973.

S. Booth, S. Chohan, J.C. Curran et al., “Whole blood viscosity and arterial thrombotic events in patients with systemic lupus erythematosus,” Arthritis and Rheumatism, vol. 57, no. 5, pp. 845-850, 2007.

G.D. Lowe, M.M. Drummond, A.R. Lorimer et al., “Relation between extent of coronary artery disease and blood viscosity,” British Medical Journal vol. 280, no. 6215, pp. 673-4, 1980.

Lacoste L, Lam JY, Hung J et al., “Hyperlipidemia and coronary disease. Correction of the increased thrombogenic potential with cholesterol reduction,” Circulation vol. 92, no. 11, pp. 3172-7, 1995.

Y. Tsuda, K. Satoh, M. Kitadai, et al., “Effects of pravastatin sodium and simvastatin on plasma fibrinogen level and blood rheology in type II hypercholesterolemia,” Atherosclerosis vol. 122, no. 2, pp. 225-33, 1996.

Splenectomy

D.A. Robertson, F.G. Simpson and M.S. Losarsky, “Blood viscosity after splenectomy." British Medical Journal (Clin Res Ed) vol. 283, no. 6297, 573-5, 1981.

S.E. Crary and G.R. Buchanan, “Vascular complications after splenectomy for hematologic disorders,” Blood vol. 14, no. 2861, pp. 2861-8, 2009. 

C.D. Robinette and J.F. Fraumeni Jr., “Splenectomy and subsequent mortality in veterans of the 1939-45 war,” British Journal of Haematology, vol. 145, no. 6, pp. 728-32, 2009.

R.F. Schilling, R.E. Gangnon and M.I. Traver MI, “Delayed adverse vascular events after splenectomy in hereditary spherocytosis,” Journal of Thrombosis and Haemostasis, vol. 6, no. 8, pp. 1289-95, 2008.

Hyperfibrinogenemia

T. Koster, F.R. Rosendaal, P.H. Reitsma et al., “Factor VII and fibrinogen levels as risk factors for venous thrombosis. A case-control study of plasma levels and DNA polymorphisms—the Leiden thrombophilia study (LETS),” Thrombosis and Haemostasis vol. 71, no. 6, pp. 719-22, 1994.

M.N. Mishra, R. Kalra and S. Rohatqi, “Clinical profile, common thrombophilia markers and risk factors in 85 young Indian patients with arterial thrombosis,” Sao Paulo Medical Journal, vol. 161, no. 6, pp. 384-8, 2013.

E. Ernst and K.L. Resch, “Fibrinogen as a cardiovascular risk factor: a meta-analysis and review of the literature,” Annals of Internal Medicine vol. 118, no. 12, pp. 956-63, 1993.

W. Koenig, “Fibrin(ogen) in cardiovascular disease: an update,” Thrombosis and Haemostasis vol. 89, no. 4, pp. 601-9, 2003.

J. Levenson, P. Giral, M. Razavian et al., “Fibrinogen and silent atherosclerosis in subjects with cardiovascular risk factors,” Arteriosclerosis, Thrombosis, and Vascular Biology, vol. 15, no. 9, pp. 1263-8, 1995.

J. Danesh, R. Collins, R. Peto et al., “Haematocrit, viscosity, erythrocyte sedimentation rate: meta-analysis of prospective studies of coronary heart disease,” European Heart Journal, vol. 21, pp. 515-20, 2000.

P.W Kamphuisen, J.C Eikenboom, H.L. Vos et al., “Increased levels of factor VIII and fibrinogen in patients with venous thrombosis are not caused by acute phase reactions,” Thrombosis and Haemostasis vol. 81, no. 5, pp. 680-3, 1999.

Lipoprotein(a)

M.B. Boffa and M.L. Koschinsky, “Lipoprotein (a): truly a direct prothrombotic factor in cardiovascular disease?,” Journal of Lipid Research vol. 57, no. 5, pp. 745-57, 2016.

F. Dentali, V. Gessi, R. Marcucci et al., “Lipoprotein(a) as a risk factor for venous thromboembolism: a systematic review and meta-analysis of the literature,” Seminars in Thrombosis and Hemostasis, vol. 43, no. 6, pp. 614-20, 2017.

B.G. Nordestgaard, M.J Chapman, K. Ray K et al., “Lipoprotein(a) as a cardiovascular risk factor: current status,” European Heart Journal vol. 31, no. 23, pp. 2844-53, 2010. 

Thrombophilias

A. Milgrom, K. Lee, M. Rothschild et al., “Thrombophilia in 153 patients with premature cardiovascular disease ≤ age 45,” Clinical and Applied Thrombosis/Hemostasis, doi: 10.1177/1076029617703481, [Epub ahead of print], 2017.

S. Vig, A. Chitolie, D. Bevan et al., “The prevalence of thrombophilia in patients with symptomatic peripheral vascular disease,” British Journal of Surgery, vol. 93, no. 5, pp. 577-81, 2006.

C.J. Glueck, J. Munja, D. Aregawi et al., “Thrombophilia-hypofibrinolysis and atherothrombotic cardiovascular disease < or = age 45 years,” Translational Research, vol. 150, no. 2, pp. 93-00, 2007.

Antiphopholipid Syndrome

M.T. Corban, A. Duarte-Garcia, R.D. McBane et al., “Antiphospholipid syndrome: role of vascular endothelial cells and implications for risk stratification and targeted therapeutics,” Journal of the American College of Cardiology, vol. 69, no. 18, pp. 2317-30, 2017.

S. Nityanand, C. Bergmark, U. de Faire et al., “Antibodies against endothelial cells and cardiolipin in young patients with peripheral atherosclerotic disease,” Journal of Internal Medicine, vol. 238, no. 5, pp. 437-43, 1995.

R.W. Lee, L.M. Taylor Jr, G. Landry et al., “Prospective comparison of infrainguinal bypass grafting in patients with and without antiphospholipid antibodies,” Journal of Vascular Surgery, vol. 24, no. 4, pp. 524-31,1996.

E.Y. Lam, L.M. Taylor Jr, G. Landry GJ et al., “Relationship of antiphospholipid antibodies and progression of lower extremity arterial occlusive disease after lower extremity bypass operations,” Journal of Vascular Surgery vol. 33, no. 5, pp. 976-82, 2001.

Sticky Platelet Syndrome

E.P. Frenkel and E.F. Mammen, “Sticky platelet syndrome and thrombocythemia,” Hematology and Oncology Clinics of North America, vol. 17, no. 1, pp. 63-8, 2003.

E.F. Mammen, M.I. Barnhart, N.R. Selik et al., “'Sticky platelet syndrome': a congenital platelet abnormality predisposing to thrombosis?,” Folia Haematologica (Leipzig), vol. 15, no. 3, pp. 361-5, 1988.

Nephrotic Syndrome

A. Gigante, B. Barbano, L. Sardo et al., “Hypercoagulability and nephrotic syndrome,” Current Vascular Pharmacolocy vol. 12, no. 3, pp. 512-7, 2014. 

R.C. Curry Jr. and W.C. Roberts, “Status of coronary arteries in the nephrotic syndrome. Analysis of 20 necropsy patients aged 15 to 35 years to determine if coronary atherosclerosis accelerated,” American Journal of Medicine, vol. 63, no. 2, pp. 18-92, 1977.

R.J. Kallen, R.K. Brynes, A.J. Aronson et al., “Premature coronary atherosclerosis in a 5-year old with corticosteroid-refractory nephrotic syndrome," American Journal of Diseases of Children, vol. 131, no. 9, pp. 976-80, 1977.

Elevated Plasminogen Activator Inhbitor-1 Concentration and Hypofibrinolysis

I. Juhan-Vague, M.C. Alessi and P.E. Morange, “Hypofibrinolysis and increased PAI-1 are linked to atherothrombosis via insulin resistance and obesity,” Annals of Medicine , vol. 32 Suppl 1, pp. 78-84, 2000.

P.J. Levy, M.F. Gonzalez, C.A. Hornung et al., “A prospective evaluation of atherosclerotic risk factors and hypercoagulability in young adults with premature lower extremity atherosclerosis,” Journal of Vascular Surgery, vol. 23, no. 1, pp. 36-43, 1996.

G. Tsitouris, R. Stathopoulou and I. Tsevrenis, “Serum inhibition of plasmin and of plasminogen activation in thrombotic disease,” Journal of Atherosclerosis Research, vol. 7, no. 4, 425-33, 1967.

J.P. Collet, Y. Allali, C. Lesty et al., "Altered fibrin architecture is associated with hypofibrinolysis and premature coronary artery atherothrombosis,” Arteriosclerosis, Thrombosis, and Vascular Biology, vol. 26, pp. 2567-73, 2006.

Clotting Disorders

F. Bilora, C. Dei Rossi, B. Girolami et al., “Do hemophilia A and von Willebrand disease protect against carotid atherosclerosis? A comparative study between coagulopathics and normal subjects by means of carotid echo-color Doppler scan,” Clinical and Applied Thrombosis/Hemostasis vol. 5, no. 4, pp. 232-5, 1999.

F. Bilora, V. Boccioleti, E. Zanon et al., “Hemophilia A, von Willebrand disease, and atherosclerosis of abdominal aorta and leg arteries: factor VIII and von Willebrand factor defects appear to protect abdominal aorta and leg arteries from atherosclerosis,” Clinical and Applied Thrombosis/Hemostasis, vol. 7, no, 4, pp. 311-3, 2001.

F. Bilora, E. Zanon, F. Petrobelli et al., “Does hemophilia protect against atherosclerosis? A case-control study.” Clinical and Applied Thrombosis/Hemostasis, vol. 12, no. 2, pp. 193-8, 2006.

A. Tuinenberg, E.P. Mauser-Bunschoten, M.C. Verhaar et al., “Cardiovascular disease in patients with hemophilia,” Journal of Thrombosis and Haemostasis, vol. 7, pp. 247-54, 2009.

S. Biere-Rafi, A. Tuinenberg, B.W. Haak et al., “Factor VIII deficiency does not protect against atherosclerosis,” Journal of Thrombosis and Haemostasis, vol. 10, no. 1, pp. 30-7, 2012. 

R. Loeffen, Spronk HM and H. ten Cate, “The impact of blood coagulability on atherosclerosis and cardiovascular disease,” Journal of Thrombosis and Haemostasis, vol. 10, no. 7, pp. 1207-16, 2012.

Erythropoiesis-stimulating Agents and Hematocrit

D.R. Gagnon, T.J. Zhang, F.N Brand, et al., “Hematocrit and the risk of cardiovascular disease—the Framingham study: a 34-year follow-up,” American Heart Journal, vol. 27, no. 3, pp. 674- 82, 1994.

A. Phrommintikul, S. Haas, M. Elsik et al., “Mortality and target haemoglobin concentrations in anaemic patients with chronic kidney disease treated with erythropoietin: a meta-analysis,” Lancet vol. 369, no. 9559, pp. 381-8, 2007.

Aspirin

G.D. Sloop, “Decreased prevalence of symptomatic atherosclerosis in arthritis patients on long-term aspirin therapy,” Angiology , vol. 49, no. 10, pp. 827-32, 1998.

Steering Committee of the Physicians’ Health Study Research Group, "Final report on the aspirin component of the ongoing physicians’ health study," New England Journal of Medicine vol. 321, pp. 129-35, 1989.

U.S. Preventive Services Task Force, “Using aspirin for the primary prevention of cardiovascular disease,” https://www.uspreventiveservicestaskforce.org/Home/GetFileByID/768 (2009, accessed 10 June 2017).

Anabolic Steroid Abuse

G.A. Christou, K.A. Christou, D.N. Nikas et al., “Acute myocardial infarction in a young bodybuilder taking anabolic androgenic steroids: a case report and critical review of the literature,” European Journal of Preventive Cardiology, vol. 23, no. 16, pp. 1785-96, 2016.

A.L. Baggish, R.B. Weiner, G. Kanayama et al., “Cardiovascular toxicity of illicit anabolic-androgenic steroid use,” Circulation, vol. 135, no. 21, pp. 1991-2002,2017.

K. Stergiopoulos, J.J. Brennan, R. Mathews et al., “Anabolic steroids, acute myocardial infarction, and polycythemia: a case report and review of the literature,” Vascular Health and Risk Management, vol. 4, no. 6, pp. 1475-8, 2008.

P. Vanberg and D. Atar, “Androgenic anabolic steroid abuse and the cardiovascular system,” Handbook of Experimental Pharmacology, vol. 19, pp. 411-57, 2010. 
